# Genome‐wide association study for host genetic factors associated with equine herpesvirus type‐1 induced myeloencephalopathy

**DOI:** 10.1111/evj.13261

**Published:** 2020-04-06

**Authors:** Wangisa M. B. Dunuwille, Navid YousefiMashouf, Udeni B. R. Balasuriya, Nicola Pusterla, Ernest Bailey

**Affiliations:** ^1^ Maxwell H. Gluck Equine Research Center Department of Veterinary Science University of Kentucky Lexington KY USA; ^2^ Louisiana Animal Disease Diagnostic Laboratory Department of Pathobiological Sciences School of Veterinary Medicine Louisiana State University Baton Rouge LA USA; ^3^ Department of Medicine and Epidemiology School of Veterinary Medicine University of California Davis CA USA

**Keywords:** horse, equine herpesvirus myeloencephalopathy, EHM, equid herpesvirus 1, GWAS, SNPs

## Abstract

**Background:**

Equid herpesvirus (EHV‐1) infections in horses can lead to equine herpesvirus myeloencephalopathy (EHM), characterised by neurological clinical signs. The sporadic occurrence of the disease in horse herds suggests a host genetic component. A recent study reported an association between the occurrence of EHM and genetic markers on horse chromosome 6 (ECA6).

**Objectives:**

To investigate the association of EHM with genetic host factors, especially with reference to the association reported for ECA6.

**Study design:**

Genome‐wide association study (GWAS) was conducted based on 94 horses that had EHV‐1 infections and comparing the 27 developing clinical EHM to the 67 which did not.

**Methods:**

DNA samples were tested from 94 horses for 382,529 single nucleotide polymorphisms (SNPs) with the Affymetrix Axiom 670K SNP array to identify possible associations with EHM. The data analysis included tests for basic, additive, dominant and recessive modes of inheritance, haplotype associations and runs of homozygosity (ROH).

**Results:**

Results from this study did not identify significant SNPs, haplotypes or ROH associations with the development of EHM following EHV‐1 infections and excluded the involvement of a recessive genetic factor in the susceptibility to develop EHM.

**Main limitations:**

Sample size and complex phenotype.

**Conclusions:**

The results exclude the involvement of a recessive genetic factor in the susceptibility to develop clinically apparent EHM but do not have the power to exclude the involvement of other, complex host genetic factors. Furthermore, there was no association between development of EHM and genes on equine chromosome 6, as previously reported.

## 1 INTRODUCTION

Most infectious diseases exhibit a clearly delineated host specificity, both in their capacity to infect and the outcome of the infection. Host specificity is considered to be the result of co‐evolution between pathogen and host such that the pathogen is able to evade the innate and acquired immunity of the host to establish an infection. If we identify the nature of host susceptibility, we may be able to engineer a defence or treatment specific for that pathogen. A recent genome‐wide association study (GWAS) suggested an association of a single nucleotide polymorphism (SNP) in horse chromosome 6 (ECA6) with the occurrence of equine herpesvirus myeloencephalopathy (EHM).^1^ The purpose of this study was to seek confirmation of this potentially useful finding.

Equine herpesviral diseases are serious problems for horses and difficult to control. Equid alphaherpesvirus 1 (EHV‐1) is a double‐stranded DNA virus in the family *Herpesviridae* in the genus *Varicello virus*.^2^ EHV‐1 has a global distribution and at some time during their lives almost all domesticated horses will become infected with the virus resulting in a wide spectrum of clinical signs including an acute upper respiratory tract disease, abortion, neonatal death, retinopathy and severe neurological disease.^3^, ^4^ Susceptibility to EHM appears to be determined by several host factors including age, sex, physical condition, immune status and whether the infection is a primary exposure.^5^, ^6^, ^7^ It has also been reported that the neuropathogenic phenotype of EHV‐1 can result from a single nucleotide substitution within ORF30 (A→G_2254_ [amino acid N→D_752_]) in the viral DNA polymerase.^8^ However, data recently generated in our laboratory and other laboratories have demonstrated this interpretation is probably overly simplistic and the development of EHM cannot be solely explained by studying the viral determinants of virulence.^9^ An experimental infection study with a neuropathogenic EHV‐1 strain (Ab4) resulted in 0%‐35% EHM cases;^10^ similar rates have been reported for natural outbreaks in the United States and Europe (3%‐45%).^11^ These observations suggest that host genetics may play an important part in the development of EHM.

Thus, the rationale for this research is to understand the host genetic factors conferring risk of developing EHM following infection with EHV‐1. As noted below, we were unable to confirm the association of a single nucleotide polymorphism (SNP) in horse chromosome 6 (ECA6) with the occurrence of EHM.^1^

## 2 MATERIALS AND METHODS

### 2.1 Horses

We tested 94 DNA samples from horses that had EHM (n = 27) and horses that were infected with EHV‐1 but did not develop EHM (n = 67). The DNA was extracted from nasal swabs or whole blood samples that were received fresh or had been archived at −80°C. These samples were obtained from several EHV‐1 outbreaks in the United States during the time period of 2013‐2018. Of these, 55 were males and 39 were females. Of the 55 males, 13 were affected while of the 39 females, 14 were affected. Based on a Fisher's exact test, the differences in rates of EHM with respect to sex were non‐significant. Due to the small number of samples and the absence of statistical significance, distribution with respect to sex was not considered further when investigating the association. We only had ages for 21 of the cases and 23 of the controls. The average ages of the case and control horses were 10.28 and 10.5 years, respectively. Median ages among case and control horses were 9 and 7 years, respectively. The differences between the two groups were not significant and not considered further.

### 2.2 Clinical characterisation

The case definition of EHM for the selection of these samples involved horses that had clinical signs of neurological disease consistent with EHM. The neurological signs included dysmetria, ataxia and paresis of limbs, toe dragging, hypotonia of the tail and recumbency. Licensed veterinarians performed the clinical diagnosis of EHM. The horses included in the control group showed signs of EHV‐1 infection (fever, swollen legs and lymph nodes, nasal discharge and abortions) without any neurological signs. All horses used were confirmed to be EHV‐1 positive through real‐time allelic discrimination quantitative PCR (qPCR) either by us or by the institutions which provided us samples.

The herpesvirus was tested for neuropathic type {ORF30 (A→G_2254_ [amino acid N→D_752_])} in 29 of the 94 samples. Among the EHM‐affected horses, eight were tested for virus type and three had the neuropathic form. Among the control horses, 21 were tested for virus type and 4 had the neuropathic form. Based on Fisher's exact test, the difference in occurrence of EHM was not significant (*P* = .4). Therefore, due to the absence of statistical significance and the low numbers in each group, we analysed the horses according to clinical signs and without regard to the virus variant.

### 2.3 Genome‐wide association studies

Genomic DNA was extracted from samples using the QIAamp^®^ DNA blood mini kit (Qiagen) following the manufacturer's instructions. Extracted genomic DNA was stored at −80°C until further use. Genotyping was carried out using the Axiom^®^ Equine Genotyping Array (Affymetrix) which contains a total of 670 796 markers (Neogen Corporation‐GeneSeek Operations). The SNP data analysis was done using the Golden Helix SNP and Variation Suite 8.8.3 (SVS). Affymetrix quality control for the samples validated 582 598 SNPs. The call rates for all 94 horses were greater than 95%.

SNP data were removed for the following reasons: call rates below 95%, more than two alleles or minor allele frequency less than 0.05 leaving 382 529 SNPs for analyses. In one set of analyses, all 94 samples were analysed together. However, a principal component analysis indicated that draught horses, ponies, warmblood breeds and horses with unidentified breeding had a peripheral distribution relative to Thoroughbred, Paint horses and Quarter Horses. Therefore, separate analyses were done using only horses identified as Thoroughbreds, Paints and Quarter Horses resulting in 55 samples (17 cases and 38 controls). Filtering left 369 064 SNPs for these subsequent analyses. The Genotype Association Tests (Basic, Genotype, Additive, Dominant and Recessive) and Haplotype Association Tests (moving window of 6 SNPs) were conducted. In addition, tests were conducted for Runs of Homozygosity (ROH) based on regions of varying minimum sizes, numbers of samples and number of SNPs, allowing for one heterozygous position in the run. Probabilities were calculated using chi‐square or correlation tests. To control for repeated analyses, probability values were corrected using the Bonferroni correction.

## 3 RESULTS

### 3.1 Association tests

**Figure 1:**
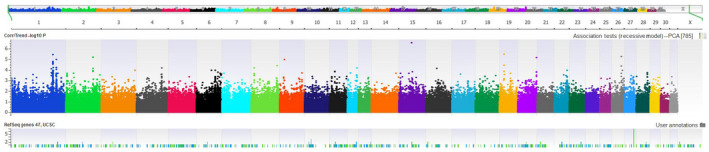
Manhattan plot of the −log10 *P* values for correlation of SNPs with EHM based on recessive model genome‐wide association study (GWAS) using 94 horses with 382,529 SNP markers analysed using the Ecab 3.0 reference genome. No significance was observed after adjusting for Bonferroni corrections

None of the association tests (Basic, Genotypic, Additive, Dominant and Recessive) identified SNPs with significant differences in distribution when comparing cases and controls. Figure 1 shows the Manhattan Plot for the investigation of the recessive mode of inheritance. Investigations for haplotype associations were conducted using sliding windows of six SNPs and, again, no associations were found. The distribution of genotypes did not indicate an influence by population substructure; however, based on observations from the principal component analyses, we did repeat the association tests using the subset of horses identified as Thoroughbred, Paint and Quarter horses based on the close relationship among those breeds. Again, no significant associations (using Bonferroni corrections) were uncovered for any of the genotype or haplotype association tests.

### 3.2 Runs of homozygosity

**Figure 2:**
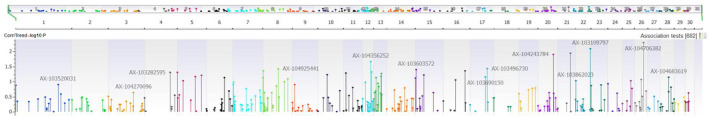
Genome plot (Ecab 3.0) of −log 10 *P* values for association test of runs of homozygosity (ROH) ROH were based on minimum five samples with a minimum homozygosity length of 400 kb with at least 60 SNPs. No significant associations were found for any ROH

Traits with recessive modes of inheritance may be missed based on recessive genotypic studies due to absence of individual SNPs which distinguish cases and controls. Therefore, analyses were conducted for runs of homozygosity. The conditions used for this study ranged from 20 samples to 5 samples required to have lengths of homozygosity from 400 to 1000 kb with an expectation of 6‐10 SNPs per kb. None of these showed statistical association of these data with EHM. A Manhattan plot for a test of recessive association this data is shown in Figure 2. Conditions for this figure were requirements for minimum of 400 kb runs with no more than one heterozygote found among 5 of the 94 horses with at least 60 SNPs. The assay identified 307 runs distributed throughout the genome with none achieving statistical significance.

### 3.3 Testing BIEC2‐946397

The single SNP identified as having a statistically significant association with EHM by Brosnahan et al^1^ was BIEC2‐946397. This SNP was included in the Affymetrix array used in this study. The locus was polymorphic in this assay with two alleles, G and A. However, the level of polymorphism was low with only five horses being heterozygous for the two alleles, two cases and three controls.

## 4 DISCUSSION

The sporadic nature for the occurrence of EHM among horses previously infected with EHV‐1 led to the speculation that some host factor may play a role in the occurrence of EHM. Brosnahan et al^1^ reported an association between a SNP and the occurrence of EHM in a manner suggesting a recessive mode of inheritance. In this current study, we examined 382 529 SNPs using a population of 94 horses (27 cases and 67 controls). In contrast to the report by Brosnahan et al,^1^ we did not find evidence for a genetic association with EHM. The power of our test was sufficient to reject a hypothesis of genetic effect by recessive alleles for this particular phenotype. We did not see any evidence for trends in the data identifying other possible modes of inheritance; however, our test does not have the power to reject other modes of inheritance such as dominant, additive or complex modes involving multiple loci because of the low number of samples in the case and control groups.

Our assay included the SNP identified in the previous work, BIEC2‐946397.^1^ The marker exhibited a low level of variation in our study and there were no statistical differences between the cases and controls for the presence of variants. While most SNPs test the same regardless of the assay used, some SNPS do not. We did not use other assays to assess the variation for the SNP and it is possible that the Affymetrix assay was not reliable for this SNP. Brosnahan et al^1^ confirmed their tests with a second assay, so we expect that our results under‐represent the variation for this SNP. Nevertheless, we did not find any evidence for linkage disequilibrium with other SNPs in the region, or with runs of homozygosity implicating genes in this region (Figures 1 and 2).

The difference between the two studies may have any of several explanations. The tests are statistical, and it may be that either of the reports failed, either in detecting^1^ or not detecting (this study) the association. There were also a few differences in the nature of the two studies that could have had an impact. In this study, all cases came from natural infections while Brosnahan and co‐workers (2018) used cases from both experimental and natural infections. The sex and ages in that study were not reported and could have had an impact on their results if the genetic effect is more likely to be manifest depending on age and sex. Both studies pooled cases of EHM regardless of type of EHV‐1 virus (wild‐type vs neuropathic form). Other differences could be related to the genetic background of the horses. In order to obtain enough horses for the GWAS study, we included horses of diverse breeds (Table S1). We do not know what breeds were used in the other study, but differences could have an impact on the statistical evaluations.

In conclusion, based on the results of this study, we do not see any evidence for a recessive allele being determinative for the development of EHM following EHV‐1 infection for any genetic locus. More complex host‐pathogen interactions are possible. Including influences by age, sex and breed. Furthermore, the phenotype used in this study, specifically occurrence of HDM, is likely the result of many factors, both genetic and environmental. Studies that focus on discrete aspects of these interactions are more likely to identify genetic host‐pathogen relationships.

## ETHICAL ANIMAL RESEARCH

Research ethics committee oversight not currently required by this journal: the study was performed on archived material collected previously during clinical procedures.

## OWNER INFORMED CONSENT

Explicit owner informed consent for inclusion of animals in this study was not stated.

## AUTHOR CONTRIBUTIONS

W. Dunuwille collected and archived the clinical samples and recorded the clinical histories, extracted genomic DNA from samples and submitted for analysis, preliminary data analysis and writing the manuscript. N. YousefiMashouf did the SNP data analysis. U. Balasuriya conceived the idea, designed the experiments and contributed to the writing of the manuscript. N. Pusterla provided a significant number of samples for this study and reviewed the manuscript. E. Bailey did the GWAS data analysis and contributed to the writing of the manuscript.

## CONFLICT OF INTEREST

No competing interests have been declared.

## Supporting information


**Table S1**

## Data Availability

The data that support the findings are available at Open Science Framework (osi.oi) https://doi.org/10.17605/OSF.IO/JH4U7.
