# Supplementary material for: Genome‐wide association study for host genetic factors associated with equine herpesvirus type‐1 induced myeloencephalopathy
Source: Equine Vet J. 2020 Apr 6;52(6):794–8. doi: 10.1111/evj.13261 (PMC7586946; doi:10.1111/evj.13261)
Supplement: Supplementary file 1 — Table S1 [file EVJ-52-794-s001.pdf]

Equine Veterinary Journal BEVA

Table S1: List of case and control samples used in this study indicating their geographical location, breed, gender, age and case/control classification. Samples were generously provided for this study by Anoka Equine Veterinary Services, Minnesota, Equine Diagnostic Solutions, Kentucky, Virginia Department of Agriculture and Consumer Services, Virginia, Alpine Veterinary Hospital, Colorado, Animal Health Clinic, Idaho, Eastern Lancaster Veterinary Clinic, Pennsylvania, Oklahoma State University-Department of Veterinary Clinical Sciences, Oklahoma, Veterinary Integrative Performance Services, Iowa, and Dr Nicola Pusterla, School of Veterinary Medicine, University of California, Davis, California.

SAMPLE	ORIGIN	CASE/CONTROL	BREED	AGE	GENDER
1	Virginia	Case	Warmblood	11	F
2	Virginia	Case	Thoroughbred	21	M
3	Virginia	Case	Quarter Horse	-	M
4	Virginia	Case	Thoroughbred	-	F
5	Virginia	Case	Thoroughbred	12	M
6	California	Case	Warmblood	-	F
7	California	Case	Warmblood	3	F
8	California	Case	Quarter Horse	-	F
9	California	Case	Quarter Horse	-	M
10	California	Case	Thoroughbred	15	M
11	California	Case	Thoroughbred	-	M
12	California	Case	Quarter Horse	15	F
13	California	Case	Warmblood	8	F
14	California	Case	Thoroughbred	15	F
15	California	Case	Paint	10	F
16	California	Case	Thoroughbred	12	F
17	California	Case	Quarter Horse	15	M
18	California	Case	Thoroughbred	15	M
20	Kentucky	Case	Thoroughbred	2	M
21	Minnesota	Case	Quarter Horse	6	M
22	Minnesota	Case	Quarter Horse	6	F
23	Minnesota	Case	Quarter Horse	17	F
24	Minnesota	Case	Not mentioned	12	F
25	Iowa	Case	Not mentioned	3	F
26	Idaho	Case	Thoroughbred	2	M
27	Pennsylvania	Case	Not mentioned	7	M
28	Wisconsin	Case	Not mentioned	9	M
29	Minnesota	Control	Not mentioned	-	M
30	Minnesota	Control	Not mentioned	-	M
31	Minnesota	Control	Not mentioned	-	F

32	Minnesota	Control	Not mentioned	-	F
33	Minnesota	Control	Not mentioned	-	F
34	Minnesota	Control	Not mentioned	-	M
35	Minnesota	Control	Not mentioned	-	M
36	Minnesota	Control	Not mentioned	-	M
37	Minnesota	Control	Quarter Horse	4	M
38	Minnesota	Control	Quarter Horse	3	M
39	Minnesota	Control	Quarter Horse	7	M
40	Colorado	Control	Not mentioned	12	F
41	Virginia	Control	Thoroughbred	16	M
42	Virginia	Control	Dutch Warmblood	4	F
43	Virginia	Control	Draft cross	17	M
44	California	Control	Quarter Horse	-	M
45	California	Control	Quarter Horse	-	F
46	California	Control	Quarter Horse	-	M
47	California	Control	Quarter Horse	-	F
48	California	Control	Quarter Horse	-	F
49	California	Control	Quarter Horse	-	F
50	California	Control	Quarter Horse	-	M
51	California	Control	Quarter Horse	-	M
52	California	Control	Quarter Horse	-	F
53	California	Control	Quarter Horse	-	F
54	California	Control	Quarter Horse	-	M
55	California	Control	Quarter Horse	5	M
56	California	Control	Crossbred	15	M
57	California	Control	Warmblood	-	M
58	California	Control	Quarter Horse	-	F
59	California	Control	Mustang	-	M
60	California	Control	Thoroughbred	10	F
61	California	Control	Crossbred	10	F
62	California	Control	Crossbred	-	M
63	California	Control	Quarter Horse	-	F
64	California	Control	Lusitano	20	M
65	California	Control	Warmblood	-	M
66	California	Control	Warmblood	-	M
67	California	Control	Not mentioned	8	M
68	California	Control	Pony	7	F
69	California	Control	Paint	12	M

70	California	Control	Crossbred	10	M
71	California	Control	Welsh pony	4	F
72	California	Control	Welsh pony	5	F
73	California	Control	Welsh pony	7	M
74	California	Control	Quarter Horse	-	M
75	California	Control	Quarter Horse	-	M
76	California	Control	Pony	-	F
77	California	Control	Thoroughbred	-	M
78	California	Control	Warmblood	-	M
79	Kentucky	Control	Thoroughbred	2	M
80	Kentucky	Control	Thoroughbred	-	F
81	Kentucky	Control	Thoroughbred	-	F
82	Kentucky	Control	Thoroughbred	-	M
83	Kentucky	Control	Thoroughbred	-	F
84	Kentucky	Control	Thoroughbred	-	M
85	Kentucky	Control	Thoroughbred	-	M
86	Kentucky	Control	Thoroughbred	-	M
87	Kentucky	Control	Thoroughbred	-	M
88	Kentucky	Control	Thoroughbred	-	F
89	Kentucky	Control	Thoroughbred	-	M
90	Kentucky	Control	Thoroughbred	-	M
91	Kentucky	Control	Thoroughbred	-	F
92	Iowa	Control	Not mentioned	4	M
93	Iowa	Control	Not mentioned	5	M
94	Iowa	Control	Not mentioned	-	F
95	Oklahoma	Control	Paint	20	M
